# Consolidation and Exacerbation of COPD

**DOI:** 10.3390/medsci6020044

**Published:** 2018-06-01

**Authors:** John R. Hurst

**Affiliations:** UCL Respiratory, University College London, London NW3 2PF, UK; j.hurst@ucl.ac.uk

**Keywords:** COPD, exacerbation, pneumonia, bacteria, consolidation

## Abstract

Twenty percent of chronic obstructive pulmonary disease (COPD) patients admitted to hospital because of an ‘exacerbation’ will have consolidation visible on a chest X-ray. The presence of consolidation is associated with higher mortality. Imperfect definitions of COPD exacerbation and pneumonia, and incomplete and imperfect diagnostic tests, have resulted in a debate about whether these episodes are best thought of as ‘exacerbation with consolidation’ or ‘pneumonia in a person with COPD’. With the current views that exacerbations are not all identical, and that they can be ‘phenotyped’ to identify episodes with different prognosis and treatment response, perhaps these episodes are best-considered a phenotype of exacerbation. Whatever the terminology, the important clinical message is to recognise that those with consolidation have higher mortality, and likely different responses to treatment.

## 1. Case History

**Figure 1:**
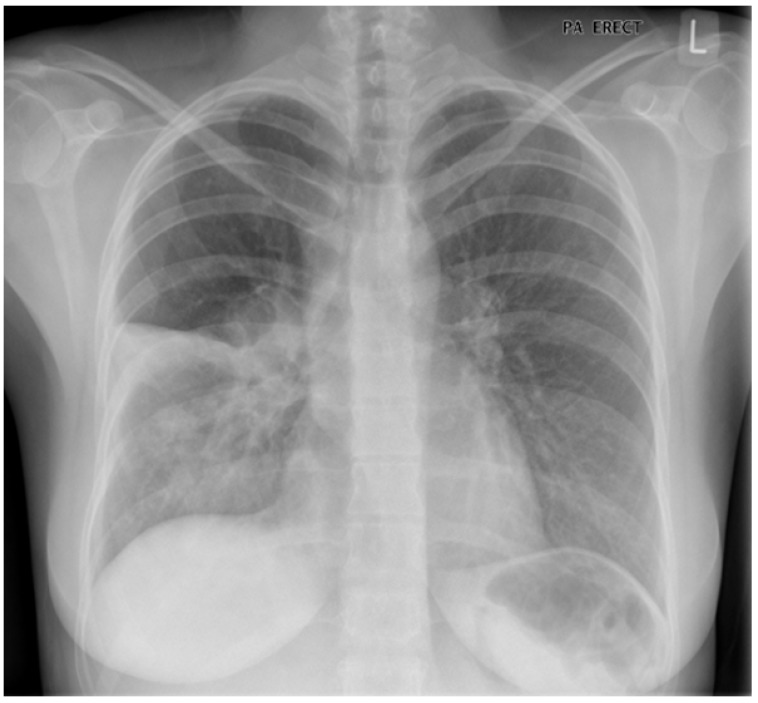
Right lower lobe consolidation in a patient with known chronic obstructive pulmonary disease (COPD): pneumonia or exacerbation?

A 67 year old woman with known chronic obstructive pulmonary disease (COPD) presents with a three-day history of increased breathlessness and a cough productive of sputum; the sputum is darker in colour than usual, and greater in volume. Her chest X-ray is illustrated below as Figure 1. The X-ray suggests consolidation in the right lower lobe. Should this be considered a pneumonia, or an exacerbation of COPD? To start to answer this question, let us first consider the definitions of these two events.

## 2. Definitions

The Global Initiative for Chronic Obstructive Lung Disease (GOLD) guideline defines a COPD exacerbation as “*an acute worsening of respiratory symptoms that result in additional therapy*” [1]. This definition contains two components: the need for a change in symptoms, and for that change in symptoms to result in additional therapy. A major criticism of the GOLD definition is that it does not reflect clinical practice: there are many causes of symptom changes in a patient with COPD that need additional therapy that are not considered COPD exacerbations—heart failure, pneumothorax and pulmonary embolus to name three. In the absence of a biomarker that can reliably differentiate exacerbation of COPD from baseline COPD [2], or exacerbation of COPD from other causes of symptom deterioration, in clinical practice an exacerbation is therefore considered a clinical diagnosis of exclusion—made when other causes of symptoms changes have been considered and where appropriate excluded through investigation [3].

There is no single definition of pneumonia. Pathological definitions focus on alveolar inflammation due to infection, whilst clinical definitions focus on a clinical syndrome of lower-respiratory tract infection with the presence of consolidation on a chest X-ray, when a chest X-ray has been performed.

## 3. The Biology of a Chronic Obstructive Pulmonary Disease Exacerbation

Our current understanding of a COPD exacerbation is that the airway inflammation that characterises COPD is further increased by stimuli such as respiratory viruses and alterations in the airway bacterial flora, resulting in bronchoconstriction, mucus plugging and ventilation-perfusion mismatch that cause the characteristic symptoms of additional breathlessness, sputum and wheeze [4]. A COPD exacerbation is therefore predominantly driven by airway, rather than alveolar infection. We know that viruses are important causes of COPD exacerbations from epidemiological studies [5], and experimental studies using human rhinovirus [6]. Understanding how alterations in bacterial flora associate with exacerbation is more complex in the era of the microbiome, but likely includes a reduction in community diversity. It is also likely that airway bacteria and viruses interact [7]. Lastly, there are epidemiological data supporting the concept that COPD exacerbations may be caused by pollutants [8].

Recently, the concept of exacerbation phenotyping has emerged [9]. This challenges the current model in which the majority of exacerbations are treated in the same way, by identifying different sub-groups of exacerbations associated with different biological pathways, and thus (perhaps) different responses to therapy. This builds on long-standing data suggesting that there is only benefit from antibiotics at exacerbation when the event is characterised by a change in sputum [10], because these events are more likely to have a bacterial aetiology. A similar concept supports the use of blood and/or sputum eosinophils to predict the response of exacerbations to respond to oral corticosteroids [11].

## 4. Exacerbation or Pneumonia: How Does the Confusion Arise?

The clinical definition of pneumonia provided above, in a patient with known COPD, does meet the GOLD definition of exacerbation. However, the clinical diagnosis of pneumonia does not meet the ‘clinical diagnosis of exclusion’ definition of a COPD exacerbation if a chest X-ray has been performed and it shows consolidation. The issue is further complicated because a chest X-ray is not routinely performed at exacerbation of COPD, outside the hospital setting and, in addition, a chest X-ray has limited sensitivity and specificity. Conceptually, with exacerbation characterised by airway infection and pneumonia by alveolar infection, it is plausible that the two may overlap and co-exist.

It might be argued that this is a semantic problem, thus the question arises as to whether the presence of consolidation on a chest X-ray at the time of an exacerbation has therapeutic or prognostic implications.

## 5. The Importance of Consolidation at Exacerbation of COPD

Because a chest X-ray is not part of routine care for most community-treated COPD exacerbations, the prevalence of consolidation at the time of a COPD exacerbation is unknown. There are data looking at the presence of consolidation in hospitalised exacerbations, but it is important to note that an exacerbation presenting to hospital, a ‘severe exacerbation’, is characterised by a more severe presentation which can arise from severe underlying COPD and/or a severe additional insult, or the presence of co-morbidities and/or a lack of social support.

Data from a European COPD Audit reported a prevalence of consolidation for hospitalised exacerbations as 19% of 14,111 cases [12]. Consolidation was associated with change in sputum at presentation, demographic factors including increased age and female sex, the presence of cardiovascular co-morbidity, and ≥2 admission to hospital in the preceding year. The presence of consolidation was also associated with increased mortality (adjusted odds ratio (OR) = 1.36 (95% confidence interval (CI) 1.20 to 1.55)). The 2014 UK National COPD Audit [13] included information on the presence of consolidation and in patient mortality from 11,513 cases. Consolidation was present in 19% and these patients had a mortality of 6.7%. The majority of patients, 81%, had no consolidation and a lower mortality at 3.6%. The prevalence of consolidation between the European and UK audits is strikingly similar.

A number of studies have examined differences between patients presenting with COPD deteriorations with and without consolidation. Huerta and colleagues [14] recruited 249 consecutive admissions to hospital with COPD, 116 of whom had consolidation. Chills, pleuritic chest pain and sputum purulence were the symptoms that best predicted consolidation, and those with consolidation also had elevated systemic inflammation as assessed by C-reactive protein (CRP, 17 vs. 7 mg/L), but milder underlying disease (higher forced expiraroty volume (FEV_1_)). Markers of systemic inflammation remained higher in those with consolidation.

Returning to the question of mortality, the presence of consolidation is included in the COPD exacerbation ‘DECAF’ prognostic score [15]. This was developed by examining features that predicted in-hospital mortality in 920 consecutive patients attending the emergency department of two UK hospitals. In addition to consolidation (C), the other variables associated with mortality and which were therefore included in the score were dyspnea (D), eosinopaenia (E), acidaemia (A) and atrial fibrillation (F). The DECAF score performed better than other scores including APACHE-II and CURB-65 score at identifying patients who died. Mortality is between 1 and 1,4% with a DECAF score (0–1) and 70% in patients with a DECAF score of 5. Results have been validated in a second study [16].

Increasingly, patients admitted to hospital with COPD undergo computed tomography (CT) scanning. It is not clear, at present, whether the presence of consolidation on CT has the same prognostic significance as consolidation on chest X-ray (CXR), but a recent study has reported that a serum CRP cutoff of 11.5 mg had a sensitivity of 91% and a specificity of 53% for CT consolidation [17].

## 6. The Importance of COPD in Patients with Pneumonia

Addressing the complimentary question of whether the presence versus absence of COPD alters prognosis in those with pneumonia, Crisafulli [18] studied 367 patients with community-acquired pneumonia, 117 of whom had COPD. Whilst the severity and prognosis as assessed by in-hospital, 30 and 90 day mortality were similar, patients with COPD tended to have a less florid inflammatory response (which wasn’t explained by differences in the use of corticosteroid drugs).

## 7. Different Therapy for Those with Consolidation

Standard therapy for a COPD exacerbation includes an increase in dose and/or frequency of short-acting bronchodilators, short-course oral corticosteroids and antibiotics when there has been a change in sputum [1]. It is important to provide appropriate support for ventilatory failure, and to attend to co-morbidities. Standard therapy for community-acquired pneumonia, outside the context of COPD, would usually comprise antibiotics to cover typical and atypical pathogens (for example a beta-lactam and macrolide combination). Whilst viruses are a common cause of COPD exacerbations, as described above, Huerta [14] did not isolate respiratory viruses in their sample of 55 exacerbations with consolidation, in which the commonest pathogen isolated was *Streptococcus pneumoniae* (in 43% of cases). The most common bacterial pathogen in the patients without consolidation was *Haemophilus influenzae*.

The Saleh study [12] reported differences in treatment between those with and without consolidation and found those with consolidation were more commonly treated with antibiotics, oxygen and non-invasive ventilation—the latter two likely reflecting greater severity, which is consistent with their greater mortality.

## 8. Summary

Twenty per cent of patients admitted to hospital with exacerbation of COPD will have consolidation visible on a chest X-ray, and this associates with higher-mortality. Imperfect diagnostics tests and definitions have created confusion in how best to refer to this: ‘exacerbation with consolidation’ versus ‘pneumonia in COPD’. As we realise that exacerbations are not all the same, and can be phenotyped, the terminology is less important than actively recognising that consolidation is a poor prognostic time, and may require different therapy. In the 21st century, exacerbation with consolidation is best considered an exacerbation phenotype.
